# Determination of Somatic and Cancer Stem Cell Self-Renewing Symmetric Division Rate Using Sphere Assays

**DOI:** 10.1371/journal.pone.0015844

**Published:** 2011-01-05

**Authors:** Loic P. Deleyrolle, Geoffery Ericksson, Brian J. Morrison, J. Alejandro Lopez, Kevin Burrage, Pamela Burrage, Angelo Vescovi, Rodney L. Rietze, Brent A. Reynolds

**Affiliations:** 1 McKnight Brain Institute, Neurosurgery department, University of Florida, Gainesville, Florida, United States of America; 2 Queensland Brain Institute, University of Queensland, Brisbane, Queensland, Australia; 3 Griffith University, Brisbane, Queensland, Australia; 4 Queensland Institute of Medical Research, Royal Brisbane Hospital, Brisbane, Queensland, Australia; 5 Institute for Molecular Bioscience, University of Queensland, Brisbane, Queensland, Australia; 6 University of Milano-Bicocca, Milan, Italy; 7 Pfizer Regenerative Medicine, Cambridge, United Kingdom; CRT Dresden, Germany

## Abstract

Representing a renewable source for cell replacement, neural stem cells have received substantial attention in recent years. The neurosphere assay represents a method to detect the presence of neural stem cells, however owing to a deficiency of specific and definitive markers to identify them, their quantification and the rate they expand is still indefinite. Here we propose a mathematical interpretation of the neurosphere assay allowing actual measurement of neural stem cell symmetric division frequency. The algorithm of the modeling demonstrates a direct correlation between the overall cell fold expansion over time measured in the sphere assay and the rate stem cells expand via symmetric division. The model offers a methodology to evaluate specifically the effect of diseases and treatments on neural stem cell activity and function. Not only providing new insights in the evaluation of the kinetic features of neural stem cells, our modeling further contemplates cancer biology as cancer stem-like cells have been suggested to maintain tumor growth as somatic stem cells maintain tissue homeostasis. Indeed, tumor stem cell's resistance to therapy makes these cells a necessary target for effective treatment. The neurosphere assay mathematical model presented here allows the assessment of the rate malignant stem-like cells expand via symmetric division and the evaluation of the effects of therapeutics on the self-renewal and proliferative activity of this clinically relevant population that drive tumor growth and recurrence.

## Introduction

Traditionally, stem cells were thought to be located only in tissues where differentiated cells were most susceptible to loss and the need for replacement great, such as the skin [1], intestinal epithelia [2] and the blood [3]. Since the adult central nervous system (CNS) was considered to lack a significant amount of neuronal death, and have no regenerative capacity, the existence of neural stem cells (NSC) seemed both unlikely, and unnecessary. However, in 1992 the existence of NSCs within the adult mammalian CNS with the ability to give rise to new neurons was demonstrated [4]. Like stem cells found in other tissues, NSCs (which line the entire ventricular neuroaxis of the adult mammalian CNS [4], [5]) exhibit the defining *in vitro* stem cell characteristics [2], [6] of proliferation, extensive self-renewal, generation of a large number of progeny, and multi-lineage differentiation potential as well as the *in vivo* characteristic of regenerating tissue after injury [4], [7], [8]. Adult stem cells represent a relatively quiescent reservoir of uncommitted cells. These cells have the ability to divide throughout the lifespan of the organism to give rise to more committed progenitor cells generating a large number of undifferentiated cells. These progenitors ultimately differentiate into lineage-restricted functional cells. Due to their ability to give rise to new cells, the factors regulating the division of stem and progenitor cells, and the differentiation of their progeny is of great interest in treating CNS disorders resulting from the loss or inappropriate functioning of cells. Therefore, the development of tools enabling stem cell-specific study represents a formidable challenge. Stem cells are difficult to visually define, as there exists no well-accepted positive marker. As a result these cells are defined based on a functional definition. While employing a functional read-out has made it possible to identify the presence (or absence) of stem cells in a population, it unfortunately prohibits the direct isolation or discrimination of stem cells from non-stem cells thereby precluding any meaningful quantitative data pertaining to their frequency and/or expansion rate.

A growing body of evidence supports the hypothesis that a population of tumor-initiating cells (TICs), which exhibit biological properties similar to normal somatic stem cells, maintains malignant tumors. TICs are postulated to reside in acute myeloid leukaemia [9], as well as in breast [10], [11], prostate [12], lung and mesenchymal tumors [13]. Importantly, neural TICs have also been isolated and found to exhibit very similar functional properties to neural stem cells [14], [15], [16], [17]. The so-called cancer stem cell model suggests that it is these stem cell characteristics that make the TICs resistant to treatment and drive the tumor recurrence. As a result these cells represent an essential target for effective anticancer therapy. Therefore development of methods investigating their biology and kinetic behavior is relevant to the design of innovative treatments targeting this specific cell population.

In the CNS, one of the methods to isolate and expand somatic and cancer stem cells is the neurosphere assay (NSA) [4], [14], [15], [18]. Of interest and attesting to its robustness, the free-floating sphere culture system is also used to study amongst others, breast cancer stem cells [11], [19]. However, while the NSA is an appropriate method to identify stem cell activity, we contend that the enumeration of spheres is not appropriate to measure stem cell frequency or expansion rate as doing so results in an overestimation [20], [21].

The current study presents the development, validation and application of a method enabling specifically quantification of somatic and cancer stem cell symmetric division rate using free floating sphere assay.

## Results

### Thought experiment

Unlike the culturing and passaging of most lines where the majority of cells survive disaggregation and go on to proliferate until the culture becomes confluent, during passaging in the NSA, the majority (>90%) of the cells die or do not further proliferate. This is supported by the fact that during one passage the majority of the dividing cells give rise to at least 256 progenies by undergoing a minimum of eight cell divisions (data not shown). This would result in a 256-fold expansion at each passage if every single plated cell were growth factor-responsive and divided 8 times. However our experiments described below show a cellular fold expansion between 1 and 20 with the different cell types we used. These data, together with published clonality data [20], [21], [22] demonstrate that less than 10% of the cells plated in the NSA contribute to the overall population expansion. Therefore only the surviving fraction of growth factor-responsive sphere-forming cells divide, form spheres, and renew the founding population. Cell death notwithstanding, during each passage, there is a geometric increase in the number of cells that are generated. Typically, if 100,000 cells are plated, greater than 90,000 of the cells die within the first 24–48 hours, leaving 10,000 cells to proliferate, form spheres and ultimately generate about 500,000 cells. This 5-fold expansion tends to be fairly consistent when passing cells over time and never shows a time-dependent escalation in fold expansion (i.e. 5-fold, 7-fold, 8-fold etc.) [23]. Moreover, each individual neurosphere line is essentially unique with regards to the fold-increase in the number of cells generated from passage to passage (some lines may show a 5-fold expansion while others a 6 or 4-fold expansion), but consistent within the particular line.

**Figure 1:**
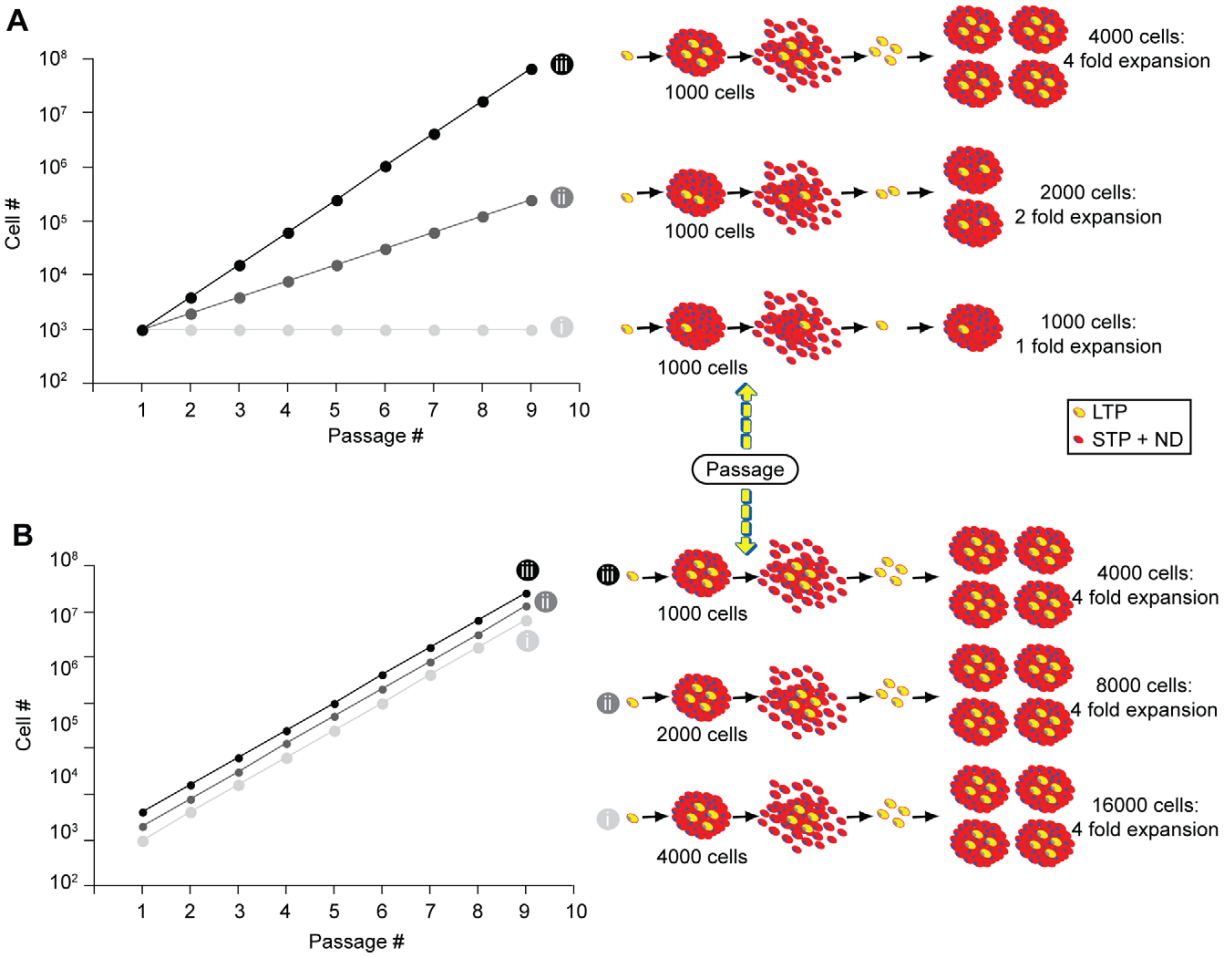
Thought paradigm. (**a**): Numerical simulation of stem/progenitor cells growth. As the number of stem cells (i.e. LTP cells) generated in a clonally derived sphere is increased, the overall fold expansion and the slope of the growth curve are increased as well. (**b**): If the numbers of stem (LTP) cells within a sphere is kept constant and the total number of cells within a sphere is doubled (ii) or quadrupled (iii), neither the fold expansion nor the slope of the growth curve are changed however the curve is elevated. LTP, long-term proliferating cells; STP, short-term proliferating cells; ND, non-dividing cells.

The ability to indefinitely serially passaged NSCs [23] and the fact that most of the cells die or stop proliferating at each passage, indicates that the population must be maintained by a long-term proliferating cell(s) (by definition a cell with stem cell features). We content that the frequency of long-term proliferating (LTP) cells (aka NSCs) will be reflected in the rate at which the population expands (i.e. fold increase from passage to passage) and this is reflected in the slope of the growth curve. In order to understand how self-renewing symmetric divisions of LTP cells affect the growth curve, consider the following thought experiment (Figure 1).

(Figure 1a) In the case of a LTP cell that generates a sphere of 1000 cells without undergoing any symmetric cell divisions, the resulting sphere will contain only a single LTP cell. Upon a subsequent passage, all the cells will again die or not participate to the culture expansion except for the single LTP cell, which survives, divides, and forms a new sphere. As we continue to passage this sphere (or population of cells) in this manner we would observe a 1-fold expansion, which is represented by a flat growth curve as seen in Figure 1a(i).
Now consider that a single LTP cell undergoes one self-renewing symmetric cell division as it generates a sphere of 1000 cells. In this case the sphere would have 2 LTP cells. Upon a subsequent passage, 998 of the cells would die and each of the two LTP cells would give rise to a sphere of 1000 cells. This doubling of the total number of spheres (and thus cells) would continue, giving a growth curve that looks like Figure 1a(ii).
Finally, if we consider that the LTP cell undergoes 3 symmetric divisions giving rise to 4 LTP cells and 996 non-LTP cells, this would produce a 4-fold expansion at each passage and a growth curve that would be expressed as in Figure 1a(iii).

(Figure 1b) If we now keep the number of LTP cells in a sphere constant (say, 4) and with each iteration change the total cells generated per sphere from 1000 (i) to 2000 (ii) to 4000 (iii), we find that the elevation of the growth curve is affected but not its slope (as seen by an unchanged fold expansion). This indicates that the total number of cells generated does influence the graph but not the slope of the growth curve.

Therefore, the cell fold expansion, represented by the slope of the growth curve, reflects the rate at which the LTP cells expand. Given that the LTP cell expansion rate is a direct indication of the numbers of self-renewing symmetric cell divisions, it follows that the fold expansion or slope can be used to predict LTP (or stem) cell self-renewing symmetric division.

### Mathematical modeling

#### Assumptions

Here we propose a mathematical model that allows one to quantify self-renewing symmetric stem cell division in tissue culture.

We break the class of all possible cells into two types (i) dividing cells and (ii) non-dividing (ND) cells. Examples of ND cells are cells that have fully differentiated or cells that have died. We further break the class of dividing cells into two subtypes, long-term proliferating (LTP) cells and short-term proliferating (STP) cells. The lifetime of the LTP cells is defined to be infinite, and in the context of an experimental setting, means that the lifetime is longer than that of the experiment. The products of a LTP cell division are assumed to be either two LTP cells (symmetric self-renewing division) or a LTP cell and a STP cell (asymmetric cell division). We assume the LTP cell differentiative symmetric division rate (LTP→STP+STP) to be null in the original growing conditions of the assay. We further assume that during the experiment time the survival and self-renewing properties of the LTP cells is definite and stable (defined by a steady state as discussed below).

The lifetime of the STP cells is defined to be finite, which in the context of an experimental setting, means that the lifetime is significantly shorter than that of the experiment. The products of a STP cell division are assumed to be any binary combination of STP cells and ND cells. It should be emphasized that this specifically excludes STP cell division from producing a LTP cell. We further assume that any dividing cell, when placed in the correct environment, will proceed through its cell cycle and eventually divide, in a manner that is independent of the presence of other cells. The products of the cell division will adhere and the cell cycle continues for all dividing cells. After time, a cluster of cells, hereafter referred to as a sphere, will have developed. Importantly, these assumptions imply that a sphere originating from a STP cell will contain STP cells and ND cells, while a sphere originating from a LTP cell will contain LTP cells, STP cells and ND cells.

Passaging neurospheres necessarily involves the dissociation of the spheres into the individual constituents (i.e. cells). A fraction of these constituent cells are then randomly sampled and seeded into a new flask containing an environment permissive for cell division (Figure 1). Here we assume that LTP cells are stem-like cells, while STP cells are more restricted progenitor cells. Hence, the long-term expansion of the population depends directly on the expansion of LTP and not STP cells. We previously demonstrated that 95% of the spheres in the NSA cannot be passed more than 4 or 6 times suggesting the majority of the spheres are derived from STP cells and that LTP cells exhibit a higher proliferative potential [20], [21]. Therefore, to accurately define a population of cells as containing stem-like (i.e. LTP) cells the overall time-course of the experiment needs to span greater than 4 to 6 passages.

#### Direct modeling of the Neurosphere Assay

After a few initial passages (corresponding to a “recovery period” if starting with freshly dissected primary tissue), the dissociating, plating, and growing of spheres in bulk culture will become consistent as the process reaches a stable state that reflects a complex equilibrium between cell survival, death, proliferation and differentiation. That is, an initial number of cells are seeded (e.g., 2.5×10^5^), which generate spheres and produce a total cell count for the flask (e.g., 1×10^6^), a portion of which are harvested (e.g., 25% are taken) and used to seed another flask for passage. The measurable quantities are the cell count at the start of the passage, *T_i_*, and the cell count at the end of the passage, *T_f_*. The fold expansion, *F*, is calculated by
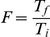



After the adaptation period of the cells to culture conditions, when the experiment has entered into the stable state, F is constant within the limits of experimental error. If the state is stable then it must also be true that the initial numbers of LTP, STP and ND cells are the same for every passage. Similarly, the final numbers must be the same for every passage. Furthermore these cell types must be undergoing the same overall fold expansion, i.e.,
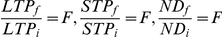



Since LTP cells can only be created from LTP cells, and every LTP cell forms a sphere, then it must also be true, that on average, there are *F* LTP cells in every LTP derived sphere. That is, the number of LTP cells created in an LTP originated sphere (defined to be *l*) must be given by, *l  =  F*.

The same analysis cannot be applied to STP cells because both LTP and STP cells produce STP cells. Similarly, the same analysis cannot be applied to ND cells. As such, data analysis becomes a matter of measuring the cell counts at the start and end of each passage, dividing the two to provide a fold expansion, then averaging the fold expansions of passages in the stable state. By definition, this average is equivalent to the number of LTP (i.e. NSCs) cells in an LTP-derived sphere. Therefore, this methodology should accurately reflect the frequency of stem-like cells in stem-like cell derived spheres.

#### LTP self-renewing symmetric division rate

The smallest time unit in the above model is a single passage. We now derive a model for the intra-passage LTP cell numbers. As noted previously, LTP cell division has two possible outcomes (i) a *self-renewing* symmetric division (LTP→LTP+LTP) or (ii) an asymmetric division (LTP→LTP+STP). We denote the probability of the first outcome by *p_ll_* and the second outcome by *p_ls_*. Since there are no other possible outcomes, the sum of these two probabilities must be unity. Let the cell cycle time of the LTP cells be denoted by *c_l_*. The probability of a symmetric cell division per unit time is thus *p_ll_*/*c_l_*. This can also be interpreted as the rate of LTP cell symmetric division and we will denote it by *K_ll_*. Since only LTP cells produce LTP cells, the rate of growth of LTP cell numbers is proportional to the current total numbers of LTP cells. It should also be noted that an asymmetric division does not change the total LTP cell numbers. The rate of growth of LTP cell numbers can thus be expressed as
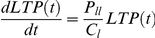



This expression can be solved to express the absolute numbers of LTP cells at a time *t*,
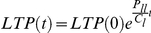



It is now possible to equate this model with the above direct model. For a passage starting at t = 0 and finishing at t = *t_f_*,
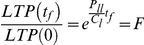



Rearranging this expression yields a method of calculating the rate of LTP cell symmetric division
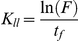



That is, the rate of LTP cell self-renewing symmetric division can be calculated by taking the natural logarithm of the fold expansion and dividing by the passage time. Therefore, changes in the fold expansion (slope of the growth curve) reflect modifications in the LTP (i.e. stem) cell frequency by variations in their self-renewing symmetric cell division rate.

### Validation of the model using the Neural Colony Forming Cell Assay (N-CFCA)

Similar to NSCs (LTP cells), progenitor (STP) cells have the ability to proliferate, and generate progeny that can be differentiated into functional cells. However, unlike stem cells, progenitor cells have a more limited proliferation potential overtime. The recently developed neural colony-forming cell assay (N-CFCA) exploits these differences in proliferative ability, enabling one to discriminate NSCs from progenitors based on the size of colonies they produce when transferred to culture [21]. Consistent with the assumption that progenitor cells exhibit limited proliferative capacity compared to stem cells, and that the size (diameter) of the colony can be used to distinguish its founder cell type, we demonstrated that large colonies (>2 mm) have a greater proliferative potential and exhibit all of the key tissue culture stem cell characteristics (extensive self-renewal, generation large number progeny and multi-lineage differentiation potential) compared to smaller colonies (which do not exhibit these stem cell criteria). Therefore, the N-CFCA provides a method to enumerate neural stem cells frequency [21].

**Figure 2:**
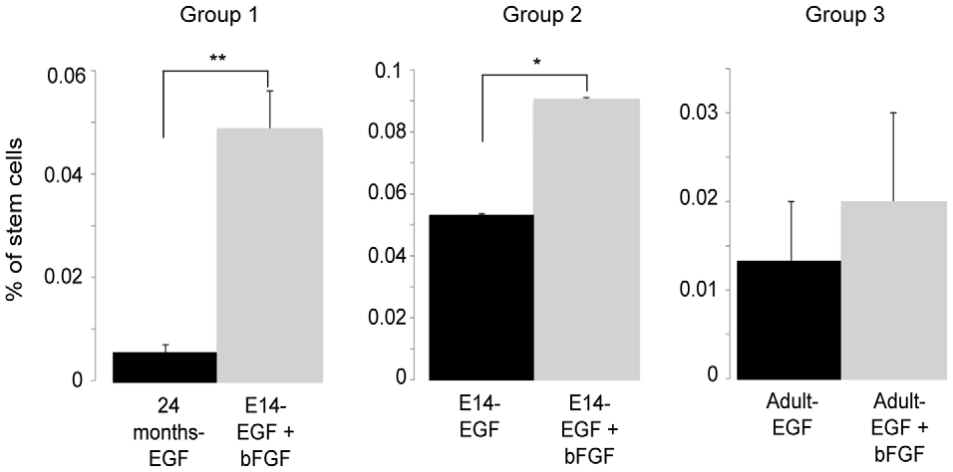
Math model validation: the Neural Colony Forming Cell Assay (N-CFCA). Cells from each group were cultured in the N-CFCA. The graphs compare in each group, the number of large colonies, greater than 2 mm in diameter (the only size range exhibiting all the key tissue culture stem cell features) obtained after 21 days *in vitro*. *p = 0.039, **p = 5.38×10^−11^, n = 15, t-test, 2 tails.

To support the mathematical modeling of the NSA with biological experiments we have compared the culturing and expansion of embryonic and adult murine NSCs in conditions that result in different growth rates (i.e. different fold expansion implying different stem cell self-renewing symmetric division rate). While the NSA model does not allow us to accurately quantify numbers of stem cells (due solely to fact that we do not know how many stem cells we started with), it does allow a comparison between groups of populations of the stem cell expansion rate, which reflects the frequency of symmetric stem cell division. In this particular case we compared the cell fold expansion of the following conditions (Table 1): (Group1) Fetal E14 NSC cultures vs. Aged adult (20 months) NSC cultures, (Group 2) Fetal E14 NSC cultures with the mitogen EGF vs. the use of EGF + bFGF, (Group 3) Adult NSC cultures with the mitogen EGF vs. the use of EGF + bFGF. From these measures the effective stem cell symmetric division rate (K*_ll_*) was derived (Table 1). Fetal stem cells cultured with both growth factors exhibited a 7.16-fold increased expansion rate compared to 24-months old stem cells cultured with EGF alone (group 1) and a 1.33-fold difference compared to the fetal cultures exposed to EGF alone (group 2). Group 3 displayed a 1.57-fold difference between the two conditions. These differences within the groups in the rate stem cells expanded was correlated to the absolute numbers of stem cells measured using the N-CFCA (Fig. 2) and the comparison results are shown in Table 2. To test the assumption that both methodologies were similar we compared the difference of their output to zero using the Student's t-test. The p value of the statistical test was 0.28 demonstrating that both assays predict a similar change in the ratio of neural stem cell number or fold expansion (aka self-renewing symmetric division rate), validating this mathematical interpretation of the NSA.

**Table 1:** Symmetric cell division rate.

Groups	1		2		3	
	E14-EGF + bFGF	24 months-EGF	E14-EGF	E14-EGF + bFGF	Adult-EGF	Adult- EGF + bFGF
F	22.2	3.1	13.6	18.1	8.0	12.6
Time (days)	7	10	7	7	7	7
K*_ll_* (divisions/day)	0.44	0.11	0.37	0.41	0.30	0.36

NSCs harvested from ganglionic eminences of E14 mice or from the periventricular region of adult mice were cultured with EGF and bFGF or with EGF alone respectively. The fold expansions (F) were compared, from which the effective rate of stem cell symmetric division (K*_ll_*) was calculated using the time for a passage.

**Table 2:** Comparison of the results obtained from the mathematical modeling (F) and the N-CFCA (stem cell number).

Comparison	Math Model (Ratio Fold Expansion)	N-CFCA (Ratio numbers of Large Colonies)
Group 1	7.16	8.98
Group 2	1.33	1.70
Group 3	1.57	1.50

Both assays predict the similar ratios between the two populations compared in each group.

### Application of the model

**Figure 3:**
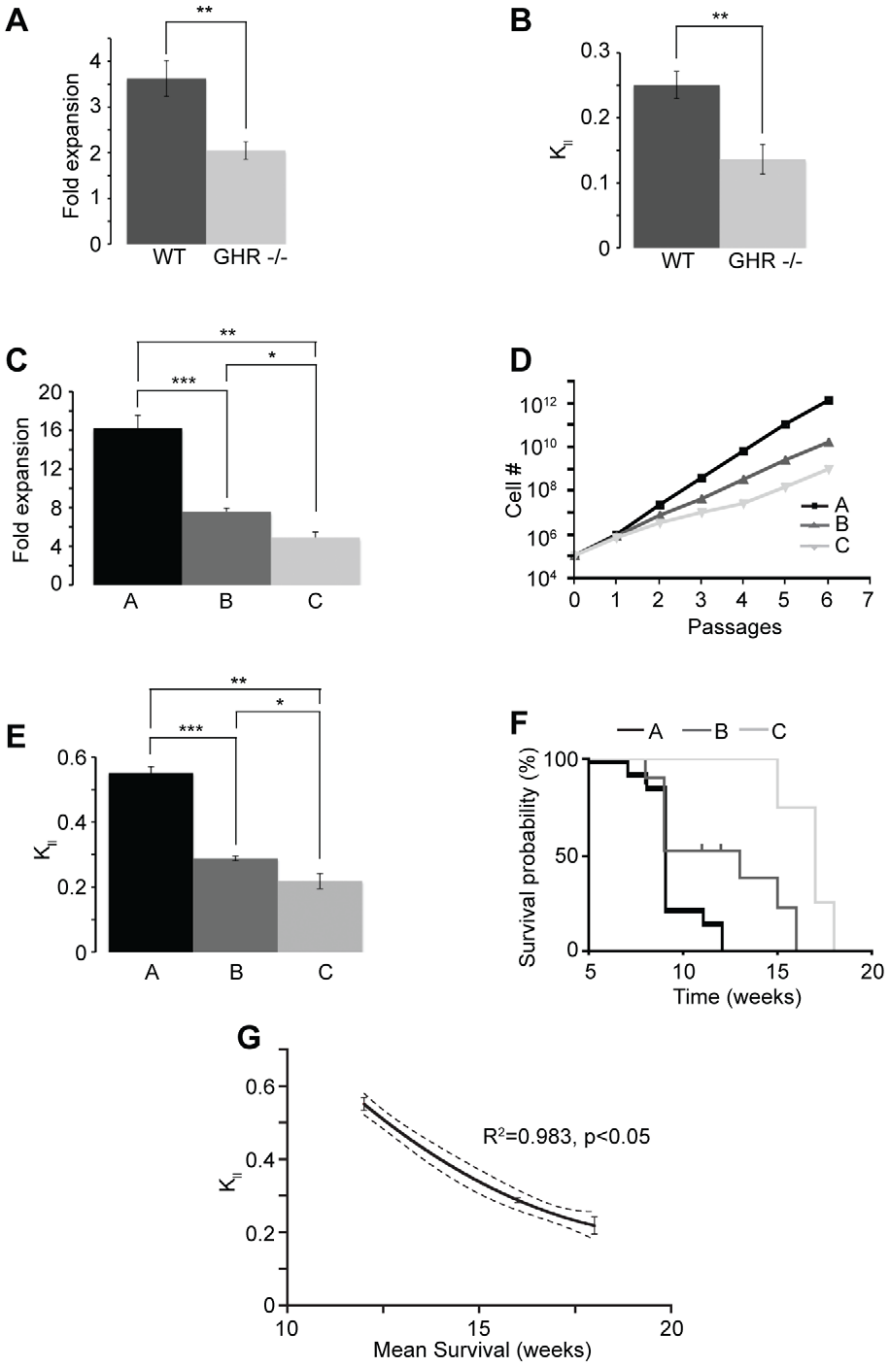
Application of the math modeling. (**a–b**) Compared to wild type animals, GHR−/− neural stem cells exhibited lower expansion rate (a, **p = 0.005, n = 7, t-test, 2 tails), which was correlated to a decreased symmetric cell division frequency (b, **p = 0.003, n = 7, t-test, 2 tails). (**c–d**) Comparison of the growth in the neurosphere assay of three human GBM samples showed distinct fold expansions (c) and slopes of growth curve (d). t-test (2 tails) was used to compare the fold expansion between lines A and B (***p = 1.4×10^−6^, n = 8–9), lines A and C (**p = 1.4×10^−5^, n = 8–6) and lines B and C (*p = 0.001, n = 9–6). (**e**) The LTP cancer cell symmetric division frequency (K*_ll_*) was derived from the fold expansion observed at each passage. t-test (2 tails) was used to compare the K*_ll_* between lines A and B (***p = 8.34×10^−12^, n = 8–9), lines A and C (**p = 1.66×10^−8^, n = 8–6) and lines B and C (*p = 0.002, n = 9–6). (**f**) Survival analysis after implantation into the striatum of NOD/SCID mice of 200,000 cells from three different tumor cell lines demonstrated an inverse relationship between the rate of symmetric division of the long-term proliferative cancer cell and the disease progression. Logrank test, p = 0.0038 comparing A to B, p<0.0001 comparing A to C and B to C. (**g**) The graph represents the mean survival after hGBM intracranial transplantation as a function of the rate LTP cancer cells undergone symmetric division (K*_ll_*). The dashed lines correspond to the 95% confidence band of the fit curve obtained by nonlinear regression (y = 2.323 – 0.2091x +0.005125x^2^). Coefficient R^2^ and p value are also shown.

We then used the model as a methodology for studying somatic stem cell self-renewing symmetric division mechanism. Using the N-CFCA, we previously showed that growth hormone receptor knock out (GHR −/−) mice exhibited significantly fewer periventricular region derived stem cells as compared to wild type animals (23±3 vs 40±3) [24]. Similarly, cultured in the NSA, compared to wild type neural stem cells, GHR −/− NSCs expanded at a significant lower rate (2.04±0.2 vs 3.63±0.4, Fig. 3a) [25] correlated to a decreased self-renewing symmetric division rate (Fig. 3b). Once again, both assays predicted the same ratios between the two populations (1.74 and 1.78 for the N-CFCA and the NSA respectively) [24]. These results suggest that growth hormone signaling controls the self-renewal of somatic neural stem cells by regulating their symmetric division rate. Pluchino and colleagues described the inhibitory effect of chronic inflammation on stem cell self-renewal [26]. This study reported that exposure of adult subventricular zone derived stem cells to inducing-inflammation Th1 cytokines caused a significant decrease in the number of NSCs measured with the N-CFCA and that this phenomenon was accompanied with a diminution of the slope of the growth curve [26]. These data further validate our mathematical interpretation of the neurosphere assay and support the model where Th1 cytokines down-regulate somatic adult stem cell expansion rate via symmetric cell division related mechanism.

The math model could also be applied to cancer biology. Several types of cancer, including those of the breast and CNS, contain cells exhibiting stem-like cell features such as long-term repopulating property [10], [14], [16], [27]. The cancer stem cell hypothesis states that tumors are hierarchically organized and maintained by a distinct subpopulation of long-term repopulating cancer stem-like cells providing therapeutic refractoriness properties. Therefore, understanding the dynamic of these cells is of great interest to understand cancer biology and to design innovative and specific treatments. We used our model to compare cancer stem-like cell (aka LTP cancer cells) self-renewing symmetric division frequency and tumor progression between several adult human glioblastoma multiforme (hGBM) cell lines grown in the Neurosphere assay conditions [16]. The three different hGBM samples analyzed (A, B and C) and generated in our laboratory exhibited distinct expansion profiles (reflected by different slopes of the curve) and LTP cancer cell symmetric division rates (Fig. 3c–e). Orthotopic transplantation of 200,000 cells from hGBM samples A, B and C into the striatum of immuno-compromised mice led to tumor formation followed by death of the animals (Fig. 3f). Importantly, the disease progression was directly and inversely correlated with the self-renewing symmetric division rate of the LTP cancer cells. This was expressed by comparing the mean survival of host animals implanted with one of the three hGBM tumor stem cell lines to the LTP cancer cell symmetric division rate (K*_ll_*) (Fig. 3g).

**Figure 4:**
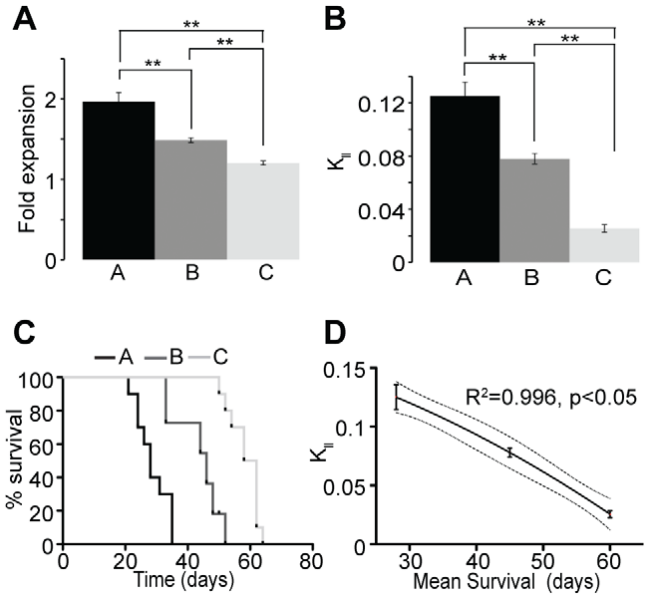
Application of the model to breast cancer. (**a–b**) Three breast cancer cell lines were cultured in the mammosphere assay. In these conditions the 3 cell lines displayed different expansion rate and LTP cell symmetric division frequency. **p<1×10^−5^, t-test, 2 tails, n = 36–48. (**c**) Tumor growth was monitored overtime between the 3 groups from which survival analysis was performed and graphed as percentage of animals that have not reach the maximal tumor size (520 mm^3^) as a function of time. Logrank test, p<0.0001 comparing the three populations to each other, n = 10. (**d**) Similar to hGBM, tumor progression after breast cancer cell s.c. transplantation was directly and inversely correlated to the symmetric division rate of the LTP cancer cell. The dashed lines correspond to the 95% confidence band of the fit curve obtained by nonlinear regression (y = 0.1743 – 0.001123x – 0.00002258x^2^). Coefficient R^2^ and p value are also shown.

In addition to brain tumors we have applied our model to breast cancer. We cultured and propagated *in vitro* three different breast tumor cell lines (KPL-1, MCF-7 and BT-474 named respectively line A, B, and C) by applying similar culture conditions used for the NSA. We used serum free medium containing human epidermal growth factor (rhEGF) and basic fibroblast growth factor (rhbFGF) allowing breast tumor cells to grow and form spherical non-adherent mammospheres [11], [19], measured the fold expansion of the breast cancer lines over six to eight passages and calculated their respective K*_ll_* (Fig. 4a–b). Our mathematical model of the mammosphere assay predicts a rate of LTP cancer cell symmetric division of 0.125 ± 0.011 for line A, 0.078 ± 0.005 for line B and 0.026± 0.003 for line C. 10^6^ cells of each breast tumor cell line were transplanted under the skin of immuno-compromised mice and formed tumors at different rate (Fig. 4c). Similar to brain tumor experiments, cell lines exhibiting higher LTP cancer cell symmetric division rate led to faster tumor progression combined with poorer survival as demonstrated in the graph comparing K*_ll_* to the mean survival of the transplanted animals (Fig. 4c–d).

Altogether these results suggest that the LTP cancer cell self-renewing symmetric division rate measured *in vitro* using Sphere Assays can be used to predict tumor progression based on the notion that increased self-renewing symmetric divisions of the LTP cancer cells will produce more tumor initiating cells resulting in a more aggressive tumor. These data also validate the potential application of our model in cancer and support the significance of the malignant LTP cell compartment in driving the expansion of the tumor and influencing the disease outcome.

**Figure 5:**
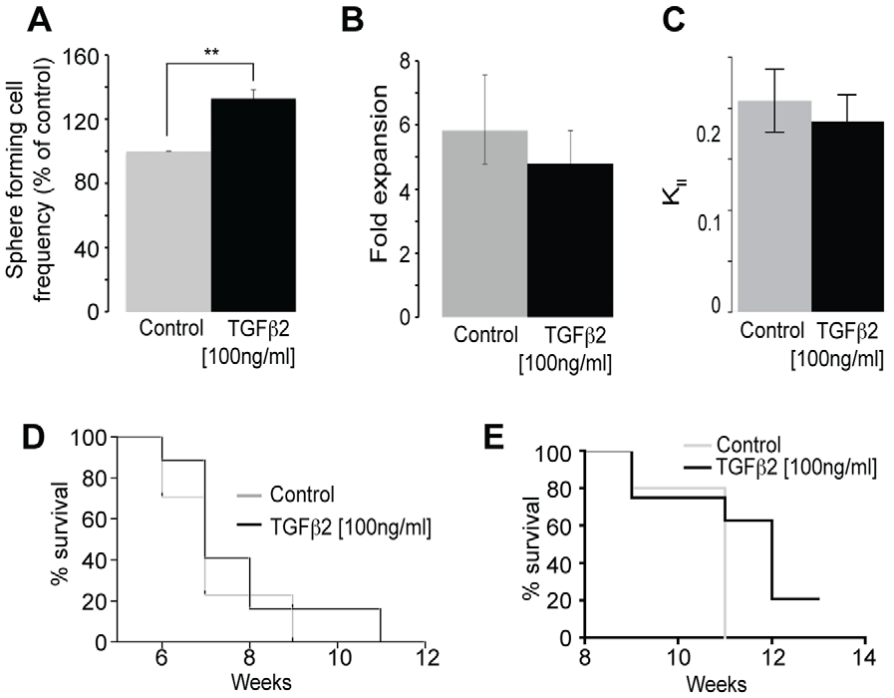
TGFβ2 does not change the long-term proliferating cell self-renewal. (**a**) Addition of TGFβ2 to hGBM cells cultured in the neurosphere assay induced an increase in the number of spheres (**p = 0.0003, n = 5, t-test). Application of 100 ng/ml of TGFβ2 to serial passed cultures did not change the expansion rate (p = 0.623, n = 4, t-test) (**b**) and the symmetric division rate (K*_ll_*) of the tumor long-term proliferative cancer cells (p = 0.645, n = 4, t-test) (**c**). TGFβ2 treatment did not enhance tumor progression of hGBM cells transplanted subcutaneously (p = 0.236, Log rank Test) (**d**) or intracranially (p = 0.158, Log rank Test) (**e**).

The model can also be used to identify agents that specifically target the LTP cancer cell population vs. those that target the STP cancer cell population. Previously we had shown that exposure of cultured hGBM to BMP4 reduces the K*_ll_* suggesting that it is targeting the LTP cancer cell population (i.e. cancer stem cells), which was confirmed by a significant reduction in the ability of BMP4 treated hGBM cells to initiate tumor formation in immunocompromised hosts [17]. Here we have extended this paradigm, using the model to identify agents that may or may not target the STP cancer cells (i.e. progenitor cells). Transforming growth factor beta 2 (TGFβ2), known primarily for its growth-inhibitory properties, is both a suppressor and promoter of tumorigenesis, producing a malignant phenotype in tumor-derived cells in culture and exhibiting high expression levels in advanced tumors [28]. Addition of TGFβ2 to hGBM tumor cell cultures resulted in an increase in the number of sphere-initiating cells (Fig. 5a). However TGFβ2 had no significant effect on the fold expansion and the rate of LTP cancer cells self-renewing symmetric divisions based on modeling of serial passage data (Fig. 5b–c). Based on the significant increase in the number of sphere forming cells, and in the absence of applying the math model, one would have interpreted the data to imply that TGFβ2 increased the proliferation of tumor initiating cells (i.e. LTP cells), while in fact it appears to not affect this population. Our data confirm the proliferative effect of TGFβ2 described in the literature [28], [29] but suggests that it drives the proliferation of STP cancer cells (or progenitor cells) as opposed to LTP cancer cells (aka cancer stem cells). Although TGFβ2 specifically increased the pool of STP cancer cells in culture, as expected, it did not enhance the tumor progression when the cells were transplanted either subcutaneously (Fig. 5d) or intracranially (Fig. 5e), as TGFβ2 did not increase the number of LTP tumor-initiating cancer stem cells. This is in line with the notion that the LTP cancer cell sub-population is associated to the ability of a population of cells to initiate and drive tumor progression and further supports a direct correlation between the rate LTP cancer cell expand *in vitro* and the aggressiveness of the tumor *in vivo*.

Together these results indicate the meaningful potential of the use of our model in studies aimed at identifying genes or molecules that regulate somatic and cancer stem cell activity.

## Discussion

The discovery of stem cells in the mature mammalian nervous system opened the door to the design and development of therapeutics to replace cells lost to injury or disease [4], [30]. Central to this approach is a detailed understanding of the mechanisms that drive NSC division and differentiation of their progeny in the context of different disease states. However, while assays exist to evaluate the division of precursor cells both *in vivo* (i.e. BrdU, Ki67, label retention) and *in vitro* (BrdU, cell counts, sphere forming assays) unraveling the contribution of stem cells from progenitor cells is difficult due to the lack of specific stem cell markers [31]. While functional assays have been widely used for assessing stem cell activity both within [7] and outside [32] the CNS, they are also fraught with difficulties related to an ambiguous read-out [20]. We have previously addressed this dilemma by developing an assay (Neural-Colony Forming Cell Assay) that is a hybrid of the tried and true classic hematopoietic precursor cells assays [32] and the NSA [4], and utilizes the extensive proliferative ability of neural stem cells relative to progenitor cells [21]. Here we report on the development, validation and application of a mathematical model that takes advantage of a peculiarity of the NSA (majority of the cells die at passage except for the growth factor responsive sphere forming cells) and allows us to estimate the relative variation in the frequency of LTP cells in this culture system. While we make the assumption that LTP cells are equivalent to NSCs, this is not without warrant, given that this feature is a defining characteristic, distinguishing stem cells from other types of proliferating precursors [21], [33], [34], [35]. We also contend that the model provides a sensitive meaningful read-out of self-renewing symmetric NSC division, as expansion of this population is dependent on this mode of division. Practically, the model can be used as a metric to compare the effects of genetic and epigenetic influences on self-renewing symmetric somatic NSC and cancer stem cell division, thereby allowing the identification of agents that can be used to increase and decrease stem cell numbers, respectively.

The neurosphere assay has been shown to be useful for somatic and cancer stem cell studies (for review [18]). The specific molecular and biochemical dissection of stem cell self-renewal and differentiation mechanisms have been hampered due to the heterogeneous nature of the neurosphere assay. Thus the free-floating culture system contains a mixed population of cells at various stages of commitment with this heterogeneity increasing with sphere size. To overcome the cellular complexity observed in this assay, adherent culture systems have been employed to isolate and expand somatic and cancer stem cells [36], [37] with the intent of providing a less heterogeneous population [38]. Notwithstanding, our mathematical modeling overcomes the barriers created by the heterogeneous cellular composition within the NSA by using a simple algorithm that enables rigorous and specific assessment of the behavior of the stem cell compartment based on bulk population analysis.

To explore and understand the complexity of a biological system and to overcome the inherent limitations of biological experiments, mathematical modeling and computer simulation approaches are widely used in system biology [39]. Different models have been proposed to provide tools to test key mechanisms at the cellular level that link the somatic/tumor stem cell compartment to tissue function and dysfunction. In the field of cancer, math modeling is not a new concept and has been used since the 1950's [40]. For example, Boman and colleagues described a kinetic model of a colonic crypt with the hypothesis that tumor formation in the colon is governed by crypt stem cell pool overpopulation via an increase in symmetric division [35], [41]. They proposed three distinct compartments - the stem cell fraction with a theoretical unlimited self-renewal capacity, the progenitor pool which displays limited renewal capacity and finally the differentiated compartment, with cells having no proliferative ability and a finite life span. The paradigm considers that stem cells are at the top of the cellular hierarchy and that the total population size remains stable overtime in normal colonic crypt, consistent with steady state conditions. The central tenet of this model is that the long-term renewable population (aka stem cells) assures tissue homeostasis and that only changes in the dynamic of the stem cell pool, via perturbation of their symmetric division rate, can account for the biologic characteristic of colorectal cancer development and progression [35]. Similarly, supported by numerical simulation, Dingli *et al*. modeled the response to treatments regarding the hierarchy and dynamic of the physiological or neoplastic hematopoietic system involving somatic and cancer stem cells. They tested several therapeutic scenarios demonstrating the critical need to target specifically the stem cell load for successful therapy [40]. As opposed to these examples of theoretical models testing the influence of hypothetic scenarios that involved kinetic changes in the stem cell pool using numerical simulation, our model is a mathematical interpretation of experimental observations enabling effective measurement of a specific cellular compartment (i.e. LTP cells) known to govern tissue homeostasis or oncogenesis.

The ability to predict LTP cell symmetric division rate using the sphere assay math model was experimentally tested using the NCFCA. This assay was chosen due to its aptitude to quantify specific changes in the neural stem cell compartment based on the notion that progenitor cells show a restricted proliferative potential compared to stem cells that have a more unlimited proliferative ability. Hence, these two populations can be distinguished based on the size of the colony they generate. Using this methodology we were able to correlate a comparison of the actual stem cell frequency (N-CFCA) (fig. 2) with stem cell expansion rate (related to symmetric division rate, in our math model) under different experimental circumstances (Table 1). We found corresponding, nearly identical (non-significant) changes in the size of the stem cell pool in both assays (Table 2). Although we validated our mathematical modeling using an *in vitro* assay, its direct correlation to physiological *in vivo* measurements (fig. 3 and 4) supports that the model is rooted in biologically reasonable assumptions. Due to its simplicity and robustness the mathematical interpretation presented in this manuscript provides meaningful and compelling information about stem cell frequency and the rate these cells expand via symmetric division. As stem cells fundamentally contribute to tissue homeostasis in adults and because stem-like cells within some cancers are hypothesized to be a critical component of malignancy, validation of our model enabling direct assessment of somatic/cancer stem cell dynamics provides metric to better understand the biology of stem cell division. For instance, in validating the model we assessed the effects of mitogens (EGF and FGF) and age on symmetric NSC divisions. Our model predicted 57% increase in expansion rate when adult mouse neural stem cells were cultured with both EGF and bFGF compared to EGF alone (Table 1, group 3). These changes were confirmed by a 50% increase in neural stem cell frequency measured with the NCFCA (fig. 2, group 3). Similarly the math model prediction of a 87% decrease in neural stem cell expansion rate as a process of aging (Table 1, group 1), which was confirmed by the 89% decrease in neural stem cell frequency in the 24-months cultures compared to the embryonic cultures (fig. 2, group 1). Additionally, data mining in the current scientific literature corroborated the relationship between the output of the NCFCA and the stem cell expansion rate obtained using the NSA, hence further supporting the validity of our math model [26]. Not only providing a renewable source of cells for neurodegenerative disease studies, the NSA presents a suitable system for pharmaceutical and neurotoxicological screening aiming at designing new therapeutic approaches to target the self-renewal capacity that is essential for controlling the stem cell pool. Finally, measuring symmetric cancer stem cell (i.e. LTP cancer cells) division provides a tool to study tumor biology as well as to test effect of drugs on this particular cellular compartment that may be responsible for tumor resistance and driving long-term tumor growth (fig. 3 and 4) [15], [33], [42], [43], [44], [45], [46].

Validation of the math model supports use of this methodology as a tool that can be used for the discovery of exogenous signaling agents and endogenous genetic elements that specifically or generally regulate the stem cell compartment. Understanding, from a functional point of view, stem cell regulatory signaling provides bases for rational drug discovery aimed at treating diseases that result in cell loss or disorders that can benefit from the generation of new cells. In addition, aging is known to adversely affect the stem cell pool outside of the CNS, identifying pharmaceuticals and nutraceuticals that can preserve the functional aspects of the NSC compartment may have an important outcome in reducing the burden of age-related CNS degeneration. In addition, our model may find application in cancer biology. It is becoming generally accepted that many solid tissue cancers contain cells exhibiting stem cell characteristics and that this population and their unique properties may contribute to tumor progression and treatment resistance. Enumerating this population and quantifying the effect(s) of treatment is difficult as stem cells (both normal and malignant) are defined by function. While single and combination of markers have been useful, their validity, when it comes to accurately defining the entire cancer stem cell pool, has been challenged [47]. Methodologies that are able to functionally measure symmetric cancer stem cell (LTP) division under a variety of experimental conditions will benefit the targeting of this component, which is important for cancer stem cell expansion. In this study we positively correlate the LTP cell expansion rate with GBM and breast tumor progression *in vivo*, and demonstrate how the Sphere Assay mathematical model can be used as readout for agents that specifically reduce or eliminate the tumor stem cell pool. Hence, our method can be used to detect and grade the occurrence of the LTP cancer cells and to study their contribution to the maintenance and therapy resistance of the tumor. Hence our modeling not only provides insight into cell kinetics mechanism leading to tumor growth, but as well has significance for elaboration of new approaches toward cancer treatment.

## Materials and Methods

### Ethics Statement

Animal studies were approved by the University of Florida Institutional Animal Care and Use Committee (IACUC, Permit Number: 2008-01502) and the University of Queensland Animal Ethics Committee (UQAEC, Permit Number: QIMR-P1159).

The use of human samples in this study was approved by the University of Florida Institutional Review Board (IRB Project # 127-2009). Written informed consent was provided by all participants.

### Mouse neural stem cell culture

Neural stem cells harvested from ganglionic eminences of E14 wild type mice or from the periventricular region of adult mice (wild type or growth hormone receptor knock out) were cultured with EGF and bFGF or with EGF alone for 6 to 7 passages in the neurosphere assay as described in [21].

### The neural colony forming cell assay method

After 4 passages in the NSA, the different groups of cells [fetal E14 NSCs, aged adult (20 months) NSC and adult NSCs cultured with different combination of mitogens, EGF and bFGF or EGF alone] were cultured in the Neural Colony Forming Cell Assay as described in [21].

### Primary cell culturing and propagation of human glioblastoma

Cells were isolated from human glioblastoma (hGBMs) as described [16] and cultured in the neurosphere assay supplemented with with 20 ng/ml hEGF, 10 ng/ml basic fibroblast growth factor, and 2 µg/ml heparin. To measure the effect of TGFβ2 on the symmetric division rate of the LTP/STP cancer cells, the treatment was performed by adding 100 ng/ml of TGFβ2 to growth medium at every passage for 4 passages.

### Xenotransplantation of brain tumor cells

The tumorigenicity of hGBM cells was measured in vivo after intrastriatal or subcutaneous injection (s.c.) using non-obese diabetic/severe combined immunodeficient mice (NOD/SCID). Details of the procedure are described in the supplemental data (Materials and Methods S1).

### Propagation and Culture of Mammospheres from Established Breast Cancer Cell Lines

First, our breast cancer cell lines (A = KPL-1, B = MCF-7 and C = BT-474) were cultured in adherent/monolayer conditions [48]. For culturing cells as spheres we have used the mammosphere assay based upon culturing neural stem cells in serum-free medium containing recombinant human epidermal growth factor (rhEGF) and basic fibroblast growth factor (rhbFGF) [11], [19]. Mammosphere culture protocol is detailed in the supplemental data (Materials and Methods S1).

### Breast cancer cell transplantation

6–8 week old NOD.Cg-*Rag1^tm1Mom^Il2rg^tm1Wjl^*/SzJ mice (The Jackson Laboratory, Bar Harbor, Maine, USA) were used for the subcutaneous (s.c.) injections of breast cancer cells. Transplantation paradigm is described in supplemental data (Materials and Methods S1).

## Supporting Information

Materials and Methods S1 (DOC)
