# Supplementary material for: Determination of Somatic and Cancer Stem Cell Self-Renewing Symmetric Division Rate Using Sphere Assays
Source: PLoS One. 2011 Jan 5;6(1):e15844. doi: 10.1371/journal.pone.0015844 (PMC3016423; doi:10.1371/journal.pone.0015844)
Supplement: Materials and Methods S1 — (DOC) [file pone.0015844.s001.doc]

**Materials and Methods S1**

**Xenotransplantation of brain tumor cells:**

All research was approved by the local animal ethics committee - The University of Queensland Animal Ethics Committee (UQAEC) and The Institutional Animal Care and Use Committee (IACUC). Orthotopic transplantation: 2µl of cell suspension (100,000 cells per ml) were stereotaxically injected into the striatum (2mm lateral from Bregma and 3mm deep).

*Subcutaneous xenograft:* 200µl of media containing 10^6^ cells with 33% matrigel were injected s.c. on the flank of the animals. Ellipsoid volume of the tumors was measured using the formula ½ x length x width^2^.

For the TGFβ2 experiments, the cells were treated once (0 or 100ng/ml) during the passage before transplantation. Survival data and tumor measurements (520mm^3^ as maximum tumor volume) were the end points for the intracranial implantation and for the s.c. transplantation respectively.

**Propagation and Culture of Mammospheres from Established Breast Cancer Cell Lines:**

For mammosphere conditions, all cells were trypsinized from adherent culture with 0.5% trypsin-EDTA (Gibco, Invitrogen Australia Pty Limited, Mount Waverley, VIC, Australia), washed twice in PBS and passed through a 40 μm cell strainer (BD Falcon, Bedford, MA, USA) before being cultured as a single cell suspension in NSA media at a density of 50,000 cells/mL in low adherent T-25 tissue culture flasks (Nunc Thermo Fisher Scientific, Rochester, NY, USA). NSA media consists of DMEM/F12 (Gibco) containing 20 ng/mL rhEGF (R&D Systems, Minneapolis, MN, USA), 10 ng/mL rhbFGF (R&D Systems), 4 μg/mL heparin (Sigma, St. Louis, MO, USA), 10% proliferation supplement (NeuroCult®, Stem Cell Technologies Inc., Vancouver, BC, Canada), 0.15% bovine serum albumin (Sigma). All cell lines received 1% penicillin G-streptomycin solution (Gibco) and were grown at 37°C and 5% CO2. Growth curves were calculated by counting (triplicate) the total number of cells generated every 5-7 days starting from 250,000 cells plated in 5 mL of media in T-25 tissue culture flasks, followed by continued culture at 250,000 cells.

**Breast cancer cell transplantation:**

All experiments were approved by UQAEC.

Before transplantation, the mice received 60-day release 17β-estradiol pellets (Innovative Research of America, Sarasota, Florida, USA) placed s.c. in the interscapular region prior to cell injection [1]. Day 6 cultures of P2 mammospheres were made into single cell suspensions by trypsin treatment followed by trypsin inhibitor treatment and passage through 40 μm cell strainers. Mice were injected s.c. on the right flank with 10^6^ single cells resuspended in 100 μl of 1:1 matrigel (BD, Franklin Lakes, New Jersey, USA) PBS. Mice were followed every 1-3 days for assessment of tumor size. Ellipsoid volume of the tumors was measured using the same formula as for hGBM (½ x length x width^2^) and the endpoint of the experiments was determined when the tumor volume reached 520mm^3^.

**References**

1. Osborne CK, Hobbs K, Clark GM (1985) Effect of estrogens and antiestrogens on growth of human breast cancer cells in athymic nude mice. Cancer Res 45: 584-590.
